# Effect of rain gauge density over the accuracy of rainfall: a case study over Bangalore, India

**DOI:** 10.1186/2193-1801-2-311

**Published:** 2013-07-11

**Authors:** Anoop Kumar Mishra

**Affiliations:** Research Centre for Environmental Changes, Academia Sinica, 128 Academia Road, Section 2, Nankang, Taipei, Taiwan ROC 11529

**Keywords:** Precipitation, Rain gauge, Remote sensing, Satellite, Hydrology

## Abstract

Rainfall is an extremely variable parameter in both space and time. Rain gauge density is very crucial in order to quantify the rainfall amount over a region. The level of rainfall accuracy is highly dependent on density and distribution of rain gauge stations over a region. Indian Space Research Organisation (ISRO) have installed a number of Automatic Weather Station (AWS) rain gauges over Indian region to study rainfall. In this paper, the effect of rain gauge density over daily accumulated rainfall is analyzed using ISRO AWS gauge observations. A region of 50 km × 50 km box over southern part of Indian region (Bangalore) with good density of rain gauges is identified for this purpose. Rain gauge numbers are varied from 1–8 in 50 km box to study the variation in the daily accumulated rainfall. Rainfall rates from the neighbouring stations are also compared in this study. Change in the rainfall as a function of gauge spacing is studied. Use of gauge calibrated satellite observations to fill the gauge station value is also studied. It is found that correlation coefficients (CC) decrease from 82% to 21% as gauge spacing increases from 5 km to 40 km while root mean square error (RMSE) increases from 8.29 mm to 51.27 mm with increase in gauge spacing from 5 km to 40 km. Considering 8 rain gauges as a standard representative of rainfall over the region, absolute error increases from 15% to 64% as gauge numbers are decreased from 7 to 1. Small errors are reported while considering 4 to 7 rain gauges to represent 50 km area. However, reduction to 3 or less rain gauges resulted in significant error. It is also observed that use of gauge calibrated satellite observations significantly improved the rainfall estimation over the region with very few rain gauge observations.

## Introduction

Rainfall is one of the most discontinuous atmospheric parameters due to its temporal and spatial variability. Indian economy is highly dependent on agriculture. Accurate rainfall estimates are essential for agricultural purposes. Chief source of rainfall over Indian region is Monsoon. India Meteorological Department (IMD) use rain gauge based gridded rainfall product developed by Rajeevan et al. (2005), to monitor rainfall over India. Rain gauges are conventional tools to quantify area averaged precipitation over land surface. Dense network of uniformly distributed rain gauge stations are used to estimate rainfall for a particular area (Mishra et al. 2011). The problem of installing optimum rain gauge network has been the subject of research over the years. Insufficient gauge density leads to error in representing the areal rainfall of a region. It is also found that rainfall is also affected by the distance of rain gauge stations from the grid point (Bhowmik and Das, 2007). The purpose of this study is to analyse the effect of rain gauge density over the accuracy of the areal daily accumulated rainfall over a region in Bangalore. Use of gauge calibrated satellite observations to fill the gap over poor gauge density region is studied in the present paper. Variation in the rainfall observations as function of inter-gauge distances is also studied in this paper.

### Data analysis

**Figure 1:**
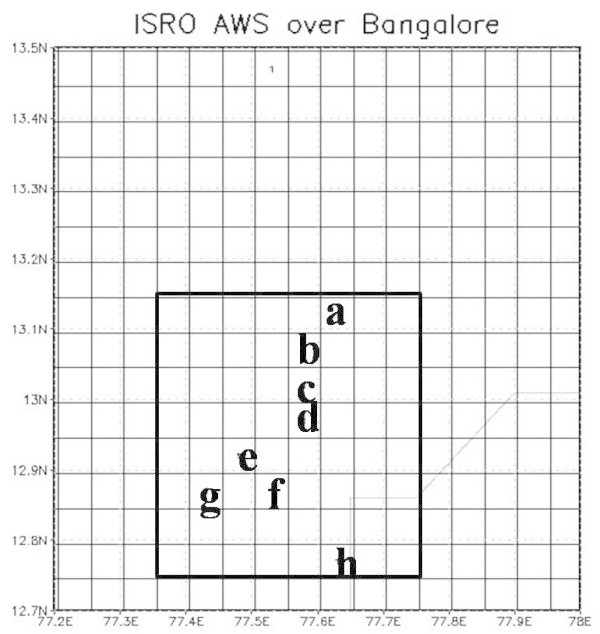
**Area of study and number of stations within it.** Alphabets represent the rain gauge stations.

In the present study, ISRO designed AWS observations are used. It has a tipping bucket rain gauge with rain measuring capacity. The data are relayed through satellite and are available through Meteorological and Oceanographic Satellite Data Archival Centre (MOSDAC). At present, there are 1098 AWS rain gauge stations over India. Distribution of rain gauges over the region is very inhomogeneous. For the present study, a region of dense network over southern part of India has been used. This region is shown in Figure 1. The density of rain gauges over this region is such that 8 rain gauges fall within an area of 50 km × 50 km box. If any station was missing in the box, then rain gauge calibrated Satellite observations were used to represent the missing station based on match-ups between the rain from the rain gauge and the Meteosat brightness temperature within the area.

Meteosat is a geostationary satellite launched in 1997 by the European Space Agency. It provides thermal infrared (TIR, 10.5-12.5 μm) and water vapor (WV, 5.7-7.1 μm) images every half an hour with a spatial resolution of 5 km. For the present study, data from 2009 to 2102 are used to study the impact of gauge calibrated satellite observations on areal rainfall estimation.

In the present study, Shepard (1968) inverse distance weighted interpolation technique has been used. It is based on the assumption that the interpolating region should be influenced most by the nearby points and less by the farthest points.

The equation is as follows:

where 'n' is the total number of rain gauge stations in the region, 'f_i_' is the rain gauge value at the i^th^ station, and w_i_ is the weight function and is given by following equation:

where p = 2, h_i_ is the distance between the interpolation point and rain gauge location.

### Rainfall variation with rain gauge spacing

**Figure 2:**
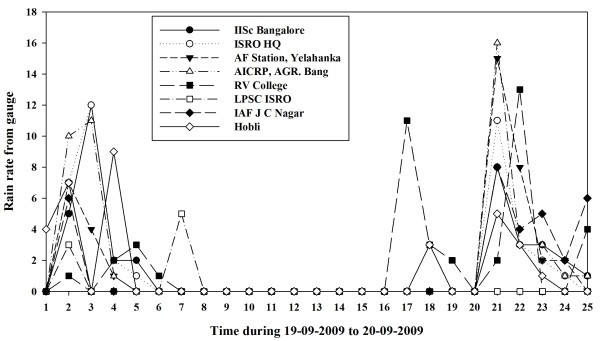
**Hourly rainfall variation shown by the gauge stations.**

Rainfall shows a great amount of variability with both space and time. A typical example is shown in Figure 2. There is considerable amount of variation in the rainfall observed by the rain gauge stations within 50 km during September 19–20, 2009. It may be noted that though stations at ISRO HQs, IISc, and Air Force Station Yelahanka are within 2–5 Km distance from each other, they show significant difference in the hourly rainfall.

361 cases of rain events were identified during rainy season of 2009–2012 to study the variation of rainfall with rain gauge spacing. Short lived intense rainfall events are defined as those with minimum hourly rainfall rate 15 mm and maximum life time of 3 hours in a day.

**Figure 3:**
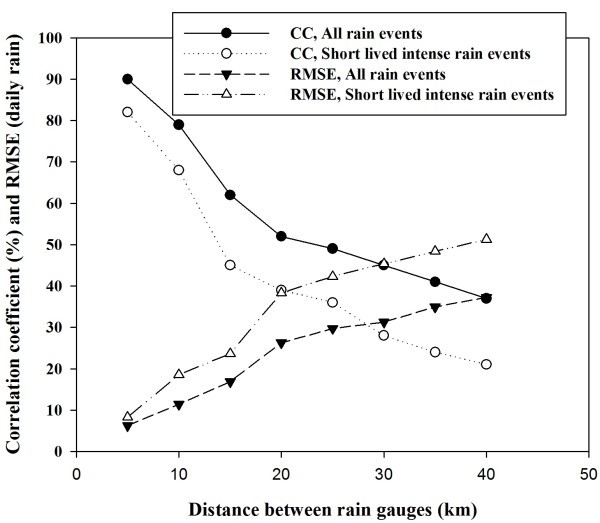
**Correlation coefficients (CC) and Root mean square error (RMSE) as function of distance between gauges.**

Figure 3, shows the effect of rain gauge spacing on the rainfall variability. It is noted from the Figure 3 that as rain gauge distance increases from 5 km to 40 km, CC decreases from 92% to 37% and rmse increases from 6.24 mm to 37.26 mm. If only short lived intense systems are considered then CC decreases from 82% to 21% while rmse increases from 8.29 mm to 51.27 mm. It may be noted that rainfall is extremely variable even inside a 50 km region. It may be observed that within 15 km rainfall variability is not abrupt. High rainfall variability is observed if the rain gauge spacing is greater than 15 km. Short lived intense rainfall events show greater variability as compared to all rain events including low but persistent rainfall cases.

### Rainfall study using gauge calibrated satellite observations

Apart from the southern part of Indian region, ISRO AWS rain gauge density over India is poor. The density over some places is such that only 1 rain gauge station (and sometimes no rain gauge) falls in 50 km × 50 km region. It is observed from the present study that rainfall values may change significantly within 15 km area. So, it is very difficult to quantify the rainfall on the basis of rain gauge observations over a region having poor rain gauge density. In this section, possibility of using rain gauge calibrated satellite observations to fill the gaps of missing rain gauge stations is analyzed. Past study (Mishra et al. 2010) shows that satellite estimates of rainfall matches well with that from rain gauge observations over well populated rain gauge area. These satellite rainfall estimates are based on a matchup between ground truth rainfall and rain signature from satellite.

**Figure 4:**
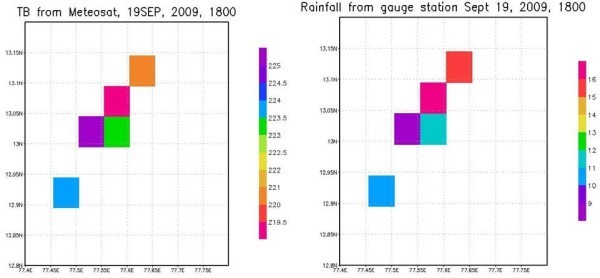
**Relation between the brightness Temp (TB) from satellite and rainfall from gauges.** Rainfall rates are in mm/h. Brightness temperature image is scaled in the decreasing order.

Figure 4, shows an example of a matchup between rainfall from rain gauge stations over area of study and brightness temperature from Meteosat satellite during September 19, 2009 at 1800 UTC. It may be observed that brightness temperature values show greater correlation with rainfall from rain gauge. A large data base using 92 cases of rainy events during 2009–2012 are used to generate a matchup between satellite observations and rain rates from rain gauge stations over area of study and this data base is used to calibrate the satellite observations from rain gauge.

**Figure 5:**
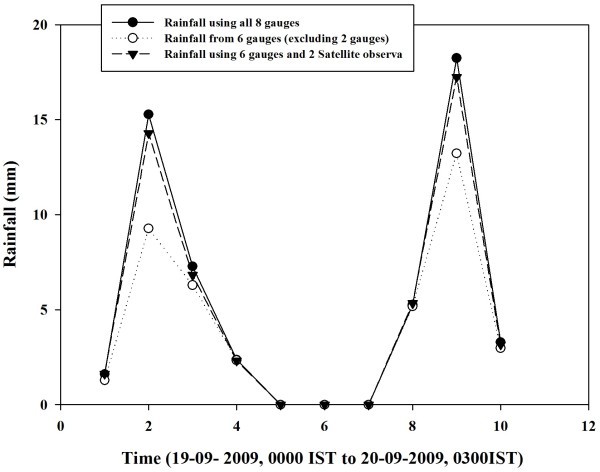
**Rainfall variation with time during 19-20 September 2009 using 8 gauges, 6 gauges and gauge plus satellite observations.**

Figure 5 shows the impact of including rain gauge calibrated satellite observations on the rainfall estimation over area of study during 19–20 September 2009. It may be observed that heavy rainfall was underestimated by using 6 rain gauges only. When the two missing rain gauge stations were filled by rain gauge calibrated satellite observations, two rainfall estimates were almost similar.

**Figure 6:**
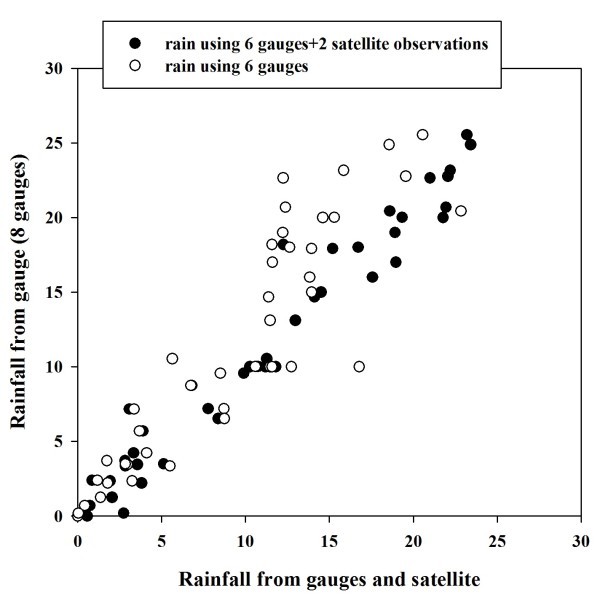
**Scatter plot between rainfall (3-hourly) using 8 gauges and rainfall using 6 gauges (empty circle) and 6 gauges plus 2 gauge calibrated satellite observations (black circle).**

Figure 6 shows the scatter diagram between rainfall using all 8 rain gauges over area of study and rainfall using 6 rain gauges. Impact of filling rain gauge station from gauge calibrated satellite observation is also shown in Figure 6. It is observed that rainfall is underestimated if two rain gauges were excluded from the analysis. If rain gauge calibrated satellite observations are used to represent the two missing rain gauge observations then rainfall estimates were improved over the area of study. It may be concluded from this study that rain gauge calibrated satellite observations can be used to supplement the rainfall information over the region having poor rain gauge density.

### Impact of rain gauge density in rainfall estimation

It is found from section 1 that rainfall in a region of 50 km box shows considerable variability. The estimate is affected by number of gauge stations in the area of study. Effect of rain gauge density over the accuracy of the rainfall estimation is studied in this section. For this purpose, total number of 274 rainy cases were considered during the years 2009–2012.

**Figure 7:**
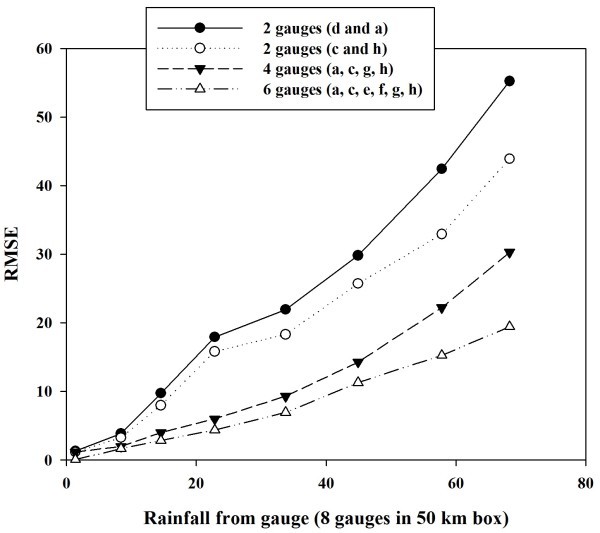
**Scatter and line plot between binned rainfall rate using all 8 gauges and rmse between rainfall using 8 gauges and that using 6, 4 and 2 gauges.**

Figure 7 shows the plot between the binned rainfall (bin width 5 mm) using 8 rain gauges and rmse between rainfall from 8 rain gauges and those using 6, 4 and 2 rain gauges. It is observed that the error increases with the increase in rainfall values. This error also increases with the decrease in the number of rain gauges used in the rainfall estimation. If the number of rain gauges are 4–6 then error is less but further decrease in the number of rain gauges resulted in significantly high error.

**Figure 8:**
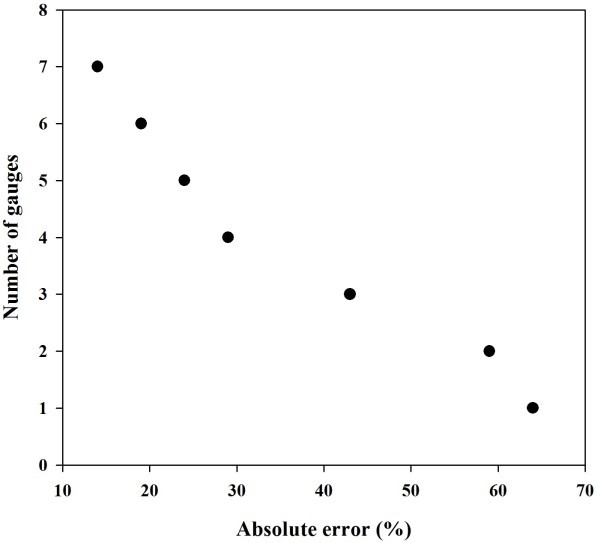
**Scatter plot between the absolute error and number of gauges.**

Figure 8, shows the effect of reduction in number of rain gauges on the absolute error in rainfall estimation. It may be observed that with 4–7 rain gauges in 50 km area, errors are less but reduction to 3 or less rain gauges resulted in high error. If single rain gauge is used to estimate the rainfall then the error increases up to 64%. It may be concluded that 4–7 rain gauges give a reasonable accuracy in rainfall estimation.

## Conclusion

In the present paper, ISRO AWS rain gauge stations over southern part of India having a good rain gauge density are used to study the effect of rain gauge density and gauge spacing on rainfall estimation. Possibilities of using rain gauge calibrated satellite observations to represent vacant rain gauge stations are also explored in this study. Significant variations are observed even among stations located within about 15 km of each other. Error increases with increase in rain gauge spacing. It may also be concluded that 4–6 rain gauges give reasonable accuracy in daily rainfall estimation in a 50 km × 50 km area. There are scopes to use rain gauge calibrated satellite observations to represent the rain gauge station in area with poor rain gauge density. The technique described here may be used to estimate the rainfall over the area having insufficient number of rain gauges. Homogeneous distribution of rain gauges having sufficient number of equally spaced gauges form a perfect network to monitor the rainfall accurately over a region.
